# The Future of Osteoarthritis Management: Plasma Exchange as a New Generation Treatment

**DOI:** 10.7759/cureus.83889

**Published:** 2025-05-11

**Authors:** Yamac Akgun, Doruk Akgün, Isik Akgun

**Affiliations:** 1 Pathology, University of Southern California Keck School of Medicine, Los Angeles, USA; 2 Pathology and Laboratory Medicine, Children's Hospital and Medical Center, Los Angeles, USA; 3 Center for Musculoskeletal Surgery, Charité - Berlin University of Medicine, Berlin, DEU; 4 Orthopedic Surgery, Gayrettepe Florence Nightingale Hospital, Istanbul, TUR

**Keywords:** apheresis therapy, arthritis and orthopaedic rheumatology, joints osteoarthritis, plasma exchange therapy, systemic immune-inflammation

## Abstract

Osteoarthritis (OA), traditionally viewed as a mechanical wear-and-tear condition, is increasingly recognized as an inflammatory disorder with autoimmune features. Despite its high global prevalence, disease-modifying treatments remain limited. Therapeutic plasma exchange (TPE) offers a novel, mechanistically targeted approach by removing systemic inflammatory mediators, pathogenic autoantibodies, and degradative enzymes such as matrix metalloproteinases. By modulating cytokines like interleukin-1 beta (IL-1β), tumor necrosis factor-alpha (TNF-α), and interleukin-6 (IL-6) and depleting cartilage-directed autoantibodies, TPE may disrupt the inflammatory and immune cascades driving joint degradation. This editorial explores TPE as a potential adjunct therapy in OA, particularly for patients with inflammatory or metabolic phenotypes. While complications such as hypotension, vascular access issues, and transient immunosuppression must be considered, the safety profile of TPE is well-established in other autoimmune conditions. Future clinical trials are urgently needed to validate efficacy, optimize protocols, and identify ideal patient subgroups. If successful, TPE could represent a paradigm shift from symptomatic relief toward disease modification in OA, with the potential to improve long-term outcomes and quality of life.

## Editorial

Osteoarthritis (OA) is the most common chronic joint disorder, affecting over 240 million people worldwide [1]. The disease disproportionately impacts older adults, but its prevalence is rising among younger populations due to increasing rates of obesity, physical inactivity, and joint injuries [1,2]. Globally, OA is a leading cause of disability, with substantial socioeconomic costs due to medical expenses, lost productivity, and caregiver burden [2].

Pathophysiologically, OA has long been considered a mechanical disease driven by cartilage degeneration, subchondral bone remodeling, and osteophyte formation [3]. However, recent advances in research reveal a far more complex interplay of biological processes [4]. Emerging evidence points to inflammation as a key player in OA progression, challenging the traditional "wear-and-tear" paradigm [4]. This shift in understanding underscores the need to move beyond symptom management and develop therapies that address the underlying mechanisms driving the disease.

Central to OA pathogenesis is the imbalance between anabolic and catabolic processes in joint tissues [5]. Mechanical stress and injury initiate a cascade of events in which chondrocytes, synoviocytes, and immune cells release degradative enzymes and inflammatory mediators [4]. Among these, matrix metalloproteinases (MMPs) are particularly significant. These zinc-dependent enzymes, including MMP-1, MMP-3, and MMP-13, degrade extracellular matrix (ECM) components such as type II collagen and aggrecan [6]. Overexpression of MMPs, driven by inflammatory cytokines, accelerates cartilage breakdown and prevents effective tissue repair. Consequently, MMP activity serves as both a marker of OA severity and a therapeutic target in efforts to mitigate joint destruction [7]. In addition to the well-established inflammatory mediators in OA, recent studies have highlighted the role of autoantibodies in the disease process, further complicating the pathophysiology of OA.

Osteoarthritis and inflammation: a paradigm shift

The identification of low-grade inflammation in OA has transformed our understanding of the disease. Key pro-inflammatory cytokines, including interleukin-1 beta (IL-1β), tumor necrosis factor-alpha (TNF-α), and interleukin-6 (IL-6), orchestrate a pathological feedback loop within the joint environment [8]. These cytokines are released by synovial macrophages, activated chondrocytes, and adipose tissue, contributing to cartilage degradation, synovial inflammation, and pain sensitization. IL-1β is a primary driver of catabolic activity in OA [8,9]. It stimulates the production of MMPs and suppresses the synthesis of ECM components like aggrecan and type II collagen, exacerbating cartilage erosion. TNF-α plays a pivotal role in the amplification of inflammation. It upregulates MMPs and other cytokines, perpetuating tissue damage and inflammation in the synovium and cartilage. IL-6 bridges systemic and local inflammation. Elevated IL-6 levels are associated with synovitis, joint pain, and radiographic OA progression, particularly in obese individuals with metabolic OA [8].

Role of autoantibodies in osteoarthritis

Although OA is not classically considered an autoimmune disease, growing evidence suggests that autoantibodies may play a role in its pathogenesis, particularly in subsets of patients with inflammatory OA phenotypes [9]. Autoantibodies targeting cartilage-specific components, such as type II collagen, aggrecan, and cartilage oligomeric matrix protein (COMP), have been identified in the serum and synovial fluid of some OA patients [10,11]. These autoantibodies are thought to contribute to joint damage by forming immune complexes that activate the complement system, leading to inflammation and degradation of cartilage. For example, anti-type II collagen antibodies can exacerbate chondrocyte apoptosis and MMP activity, intensifying cartilage breakdown. Similarly, autoantibodies against aggrecan, a critical proteoglycan in cartilage structure, are associated with increased cartilage turnover and tissue damage [11]. The presence of these autoantibodies highlights the potential overlap between OA and autoimmune processes, particularly in patients with systemic inflammation or comorbidities like obesity or metabolic syndrome.

Role of metabolic syndrome and systemic low-grade inflammation

Obesity, metabolic syndrome, and insulin resistance are increasingly recognized as significant contributors to OA development and progression, even in non-weight-bearing joints such as the finger joints. These conditions create a state of systemic low-grade inflammation, characterized by elevated levels of pro-inflammatory mediators and adipokines [12]. Adipokines such as leptin, resistin, and adiponectin link metabolic dysfunction to joint pathology. Leptin, for example, enhances the production of MMPs and pro-inflammatory cytokines, creating a vicious cycle of inflammation and tissue degradation [13]. Similarly, systemic oxidative stress and advanced glycation end products (AGEs) promote cartilage damage and subchondral bone remodeling, further exacerbating OA.

Limitations of current treatment options

Current treatment options for OA are primarily aimed at managing symptoms, but they fall short of addressing the underlying mechanisms driving disease progression. Nonsteroidal anti-inflammatory drugs (NSAIDs) are widely used to alleviate pain; however, their benefits come with significant risks, including gastrointestinal, cardiovascular, and renal side effects, particularly in older patients. Moreover, NSAIDs are ineffective in halting cartilage degradation or altering the disease trajectory [14]. Intra-articular injections, such as corticosteroids, provide temporary relief by suppressing inflammation but may paradoxically accelerate cartilage loss with repeated use. Hyaluronic acid injections, another commonly employed option, offer only modest benefits in select patients, with efficacy varying significantly across populations.

For advanced stages of OA, surgical interventions such as total joint replacement often become the only viable option, underscoring the lack of effective early disease-modifying treatments. Emerging therapies, including regenerative approaches like platelet-rich plasma (PRP) and mesenchymal stem cells, hold promise but face challenges related to standardization, accessibility, and inconsistent outcomes [14]. Similarly, biologic agents targeting key inflammatory cytokines such as IL-1β, TNF-α, and IL-6 have shown limited success, likely due to the multifactorial and heterogeneous nature of OA.

Mechanism of action of plasma exchange

Therapeutic plasma exchange (TPE), also known as therapeutic plasmapheresis, is an extracorporeal therapy designed to remove pathogenic components from a patient’s plasma. This procedure involves withdrawing blood, separating and discarding the plasma, and replacing it with a substitute solution, typically albumin or fresh frozen plasma [15]. By eliminating circulating immune complexes, autoantibodies, pro-inflammatory cytokines, and other pathological substances, TPE modulates immune responses and reduces systemic inflammation. While its applications in autoimmune and inflammatory diseases are well-established, its potential utility in OA remains underexplored.

Plasma exchange as a potential treatment for osteoarthritis

**Figure 1:**
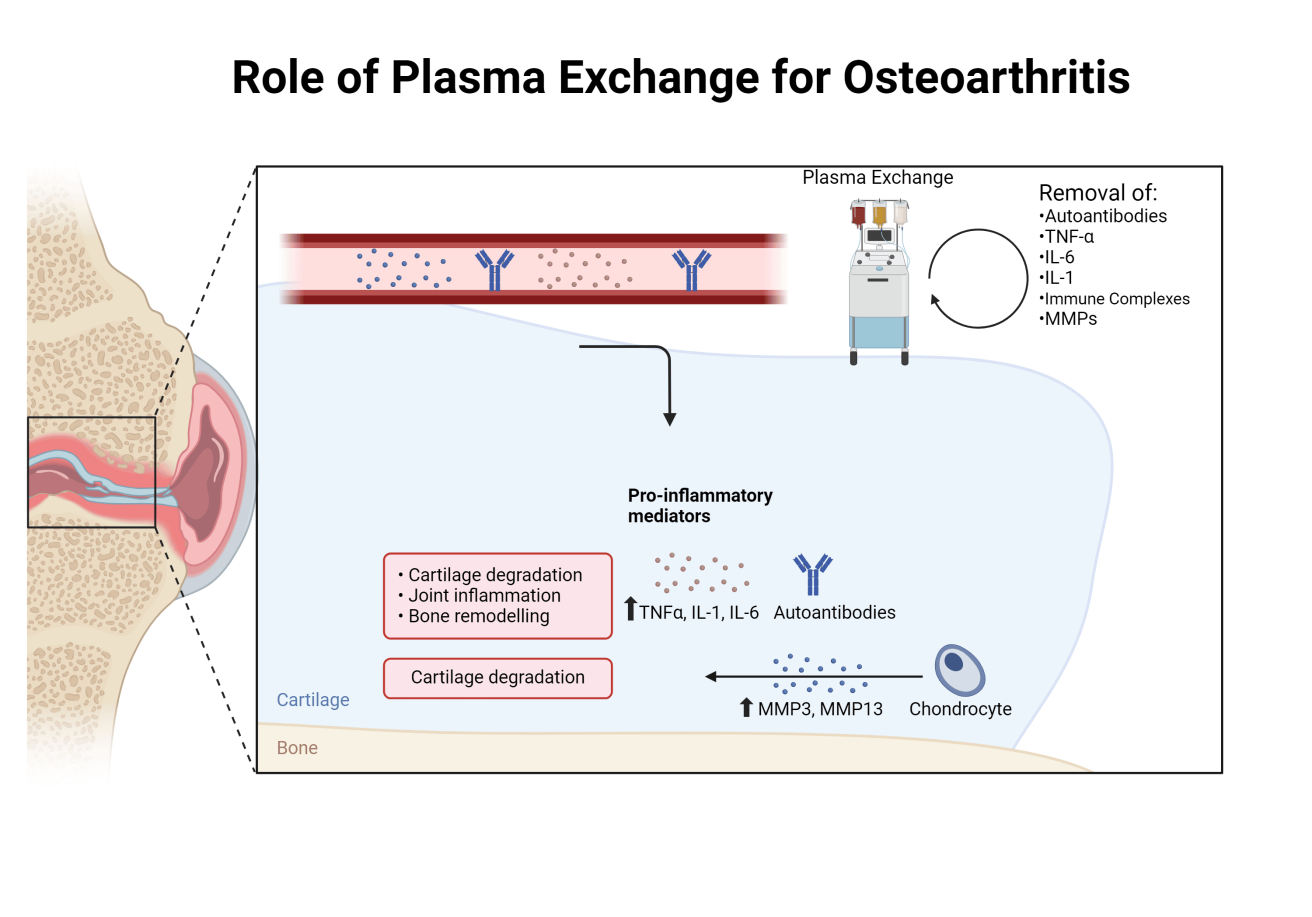
Illustration demonstrating the utilization of plasma exchange as a therapeutic strategy to address the underlying inflammatory mechanisms of osteoarthritis Credit: Illustration created with BioRender.com. Permission to reproduce this figure has been obtained through an active BioRender license held by the authors at the time of publication. TNF-α: tumor necrosis factor-alpha; IL: interleukin; MMPs: matrix metalloproteinases

The mechanism of action of TPE is particularly suited to conditions driven by immune dysregulation and chronic inflammation. Pathogenic autoantibodies, immune complexes, and pro-inflammatory mediators such as IL-1β, TNF-α, and IL-6 are known contributors to tissue damage in autoimmune diseases. TPE directly reduces their concentrations in circulation, interrupting the pathological cascade that sustains inflammation and tissue injury (Figure 1) [16]. Additionally, PE may lower the activity of degradative enzymes such as MMPs, which play a significant role in cartilage destruction in OA and other joint disorders. By mitigating the systemic inflammatory burden, TPE has the potential to address both local and systemic aspects of disease pathology [15].

Beyond its ability to clear circulating mediators, TPE may also have systemic benefits, particularly in OA patients with metabolic syndrome or obesity. These conditions are characterized by chronic low-grade inflammation, driven by adipokines such as leptin and resistin, which exacerbate joint inflammation and cartilage breakdown. TPE has been shown to reduce systemic inflammation in other conditions, offering the potential to address the metabolic-inflammatory axis in OA [17].

The rapid clearance of circulating immune complexes and damage-associated molecular patterns (DAMPs) during TPE also reduces chronic immune activation, allowing regulatory immune cells such as Tregs and M2 macrophages to re-establish an anti-inflammatory environment [16]. Emerging research suggests that the systemic reduction of inflammatory mediators following TPE can influence epigenetic regulators, such as histone acetylation and DNA methylation, further modulating gene expression profiles to favor anti-inflammatory responses [16]. The future of TPE as a therapeutic modality in OA hinges on rigorous research to refine its applications and optimize patient outcomes. Key directions include designing well-structured clinical trials to evaluate its efficacy in modifying disease progression, alleviating symptoms, and improving quality of life in OA patients with inflammatory phenotypes.
